# Role of Sex Hormones at Different Physiobiological Conditions and Therapeutic Potential in MBD2 Mediated Severe Asthma

**DOI:** 10.1155/2021/7097797

**Published:** 2021-12-14

**Authors:** Binaya Wasti, Zhifeng Chen, Yi He, Wen Tao Duan, Shao-Kun Liu, Xu-Dong Xiang

**Affiliations:** Department of Pulmonary and Critical Care Medicine, Research Unit of Respiratory Disease, Hunan Centre for Evidence-Based Medicine, The Second Xiangya Hospital, Central South University, Changsha, Hunan 410011, China

## Abstract

Sex hormone has become a “hot topic” to evaluate the hormonal therapeutic potential in severe asthma. Th17 cell is one of the main influencing factors involved in the pathogenesis of severe asthma, hence also called as kernel of severe asthma, and Th17 subtype of non-T2 asthma is less responsive (resistance) to inhaled corticosteroid (ICS), so severe in nature. Methyl-CpG binding domain protein 2 (MBD2) is overexpressed and regulates the Th17 differentiation, showing the possibility of therapeutic target in treating Th17 mediated severe asthma. Sex hormone fluctuates at the different physiobiological conditions of the human body and affects the asthma pathobiology showing its role in asthma prevalence, severity, remission, and therapy. This review briefly overviews the sex hormones, their influence in asthma at the different physiobiological conditions of human body, and MBD2 severe asthma connection with the possible therapeutic potential of sex steroids in MBD2 mediated Th17 predominant severe asthma. Male sex hormone tends to show a beneficial effect and possibly downregulates the expression of Th17 cells via regulating MBD2 through a mechanism distinct from corticosteroid treatment and guides us towards discovery of new therapeutic agent, reduces the asthma-related complications, and promotes long-term survival by lowering the risk of therapy-resistant issues of old age severe asthma.

## 1. Introduction

Asthma is a complex, chronic, heterogenous inflammatory disease, with diverse endotypes and phenotypes, characterized by airway inflammation and airway hyper-responsiveness (AHR), relieved spontaneously or by medications [1]. The T2 (T2 high) asthma is early-onset allergic, and late-onset nonallergic eosinophilic asthma, sensitive to ICS, and Th2 biomarkers are commonly used in diagnosis [2]. T2 low (non T2) asthma, also known as noneosinophilic asthma, is still obscure and is late-onset neutrophilic, paucigranulocytic, or mixed, and inflammation is driven through varieties of asthma-related inflammatory cells and may be associated with airway smooth muscle or neural dysfunction as well as comorbidities occasionally [3, 4]. T2 low asthma is generally less responsive to inhaled corticosteroid, hence also known as severe asthma.

**Figure 1:**
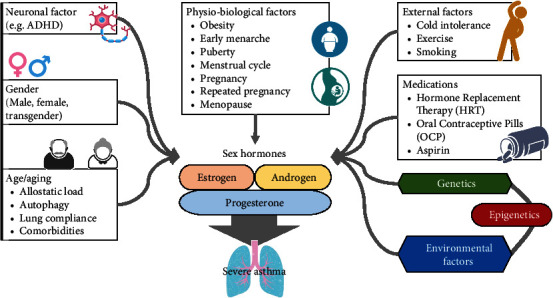
Factors associated or confound with sex hormones in severe asthma. Many internal and external factors are directly or indirectly associated with sex hormone and affect outcome of severe asthma. Gender difference is closely associated with hormonal fluctuations and associated with asthma. Allostatic load, autophagy, lung compliance, and comorbidities are aging-related factors of gender, closely associated with sex hormones, and affect asthma morbidity, mortality, and control. Physiobiological factors like obesity, early menarche, puberty, menstrual cycle, pregnancy, repeated pregnancy, and menopause are closely related to sex hormones fluctuations and asthma severity and prevalence. Personal, behavioral, and external factors, such as smoking, cold, and exercise intolerance, also affect or confound the hormonal effect and affect asthma prevalence and endotypes. Sex hormones interact with genetic and environmental factors of epigenetics to modulate inflammation and asthma severity. The interaction and association of sex hormones with internal and external factors are complex. However, understanding this complexity is beneficial for understanding the asthma pathobiology and generating a patient approach therapy.

Th17 cells and their cytokines are considered as the main influencing factors in the pathogenesis of severe asthma. That is why Th17 is also entitled as a kernel of severe asthma. Proinflammatory cytokines, oxidative stress, neuronal and hormonal responses, and epigenetic regulation are also involved in Th17 cell-mediated severe asthma, but the underlying mechanisms are still unknown.  Figure 1 shows factors associated with sex hormones affecting severe asthma.

The methyl-CpG-binding domain (MBD) family proteins determine the transcriptional state of the epigenome, can interpret DNA methylation in epigenetics [5, 6], and are equally crucial for emerging roles in immunity [5]. MBD2 binds to the target gene′s promoter region, induces posttranscriptional histone modification, changes the chromatin structure, and ultimately regulates the expression of target genes, and this is believed to be an essential critical mediator in asthma epigenetics mechanism.

It is a well-known fact that the loss of MBD2 inclines towards Th2 cellular polarization [7] and deficiency in Th17 differentiation. Our initial study in patients showed increased expression of MBD2 in Th17 mediated severe asthma from peripheral blood samples. We also found the increased MBD2 expression after stimulus differentiation in splenic CD4+ T-cells in our animal model, showing involvement of MBD2 in immunological pathogenesis of Th17 mediated neutrophilic severe asthma and differentiation of CD4+ T-cells.

Sex steroids are essential for sex differentiation and reproduction. Testosterone (TES) and other androgens, such as dihydrotestosterone (DHT), have broad immunoregulatory effects that suppress immune responses [8]. Estrogen also regulates innate immune cells and the signaling pathways [9]. The natural physiobiological conditions like menstrual cycle, pregnancy, menopause, and oral contraceptives pills (OCP), hormone replacement therapy (HRT), and epigenetics mechanism fluctuate the hormone level and affect the pulmonary outcome [10, 11].

Estrogen and progesterone are involved in the differentiation of Th17 cells. IL-17A production with IL-17A mediated airway inflammation and IL23R expression is increased by estrogen and progesterone [12]. let7f microRNA inhibits the IL-23R expression on Th17 cells [13], and estrogen and progesterone inhibit the let-7f microRNA expression, leading to the increased IL23R expression and increased IL-17A expression in Th17 cells [12]. Androgen reduces allergic airway inflammation in males and negatively regulates innate lymphoid cells 2 (ILC2) and Th2 cells [14, 15]. TES limits the neutrophilic airway inflammation by diminishing the IL-17A protein expression [15, 16]. What is the influence of sex hormones in MBD2 mediated Th17 predominant severe asthma? Does it affect MBD2 expression?

This review briefly overviews the sex hormones, their influence in asthma at different physiobiological conditions of the human body, MBD2 severe asthma association from various studies, and role of sex hormones in the MBD2 expression with the possible therapeutic potential of sex steroids in MBD2 mediated Th17 predominant severe asthma.

## 2. Sex Hormone Steroidogenesis

**Figure 2:**
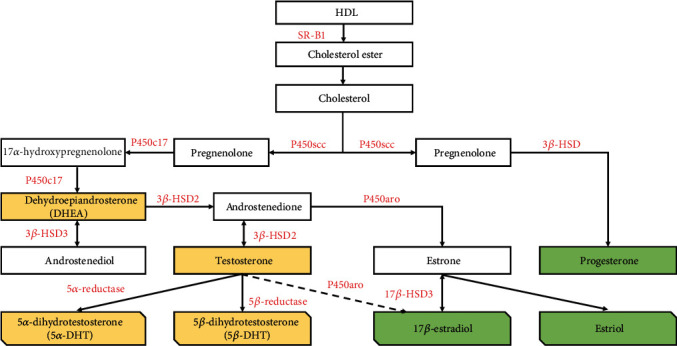
Sex hormones steroidogenesis. Cholesterol is the precursor of all steroids, including sex hormones, uptaken by plasma proteins lipoprotein receptors scavenger receptor class B member 1 (SR-BI) [17, 18]. p450scc, the side-chain cleavage enzyme, converts the cholesterol into pregnenolone [19]. Pregnenolone is metabolized to progesterone by 3*β*-hydroxysteroid dehydrogenase (3*β*-HSD), and P450c17 transforms progestogen to 17-hydroxypregnenolone and dehydroepiandrosterone (an androgen, DHEA). Hydroxylation of DHEA to androstenedione and androstenediol is mediated by 3*β*-hydroxysteroid dehydrogenase 2 (3*β*-HSD2) and 17*β*-hydroxysteroid dehydrogenase 3 (17*β*-HSD3), respectively [19]. Androstenedione and androstenediol biotransfered to TES by 3*β*-HSD2 and 17*β*-HSD3, respectively. 5*α*-dihydrotestosterone (5*α*-DHT) [20] and 5*β*-dihydrotestosterone (5*β*- DHT) [21] are the reducing TES product by 5*α*-reductase type 1 or 2 and 5*β*-reductase, respectively [20, 21]. P450 aromatase (P450aro) bioconverts androstenedione to estrone and TES to 17 *β*-estradiol [19]. Estrone is also bioconverted to 17 *β*-estradiol by 17*β*-HSD3. TES: testosterone.

Cholesterol is the precursor of all steroids, including sex hormones that primarily come from cholesterol ester, uptaken by plasma proteins lipoprotein receptors, scavenger receptor class B member 1 (SR-BI) [17, 18]. The side-chain cleavage enzyme p450scc, an essential enzyme to synthesis all steroid hormones, converts the cholesterol into pregnenolone [19]. Pregnenolone is a progestogen and can be metabolized to progesterone by 3*β*-hydroxysteroid dehydrogenase (3*β*-HSD), and P450c17 transforms this progestogen to 17-hydroxypregnenolone and dehydroepiandrosterone (an androgen, DHEA). Hydroxylation of DHEA to androstenedione and androstenediol is mediated by 3*β*-hydroxysteroid dehydrogenase 2 (3*β*-HSD2) and 17*β*-hydroxysteroid dehydrogenase 3 (17*β*-HSD3), respectively [19]. TES is the biotransformed product of androstenedione and androstenediol by 3*β*-HSD2 and 17*β*HSD3. 5*α*-dihydrotestosterone (5*α*-DHT) [20] and 5*β*-dihydrotestosterone (5*β*- DHT) [21] are the reducing TES product by 5*α*-reductase type 1 or 2 and 5*β*-reductase, respectively [20, 21]. P450 aromatase (P450aro) bioconverts androstenedione to estrone and TES to 17 *β*-estradiol [19]. Estrone is also bioconverted to 17 *β*-estradiol by 17*β*-HSD3 (Figure 2 for sex hormones steroidogenesis).

## 3. Early Childhood, Sex Hormones, and Asthma

During childhood, boys experience more frequent asthma symptoms with higher asthma prevalence than girls (ratio of 2 : 1 before the age of 5 years) [22, 23]. This ratio further increases to 4-fold until the age of 14, and the gender switch occurs to 1 : 2 as the plasma TES level increases in boys [24–27] (Table 1 shows asthma and sex hormone cycles in human life).

(Note: in boys, asthma prevalence in childhood is higher than girls, and there is a gender switch to females from puberty to adulthood as the male sex hormone and airway caliber is believed to be involved because old aged male tends to have increased asthma episodes than previous stage as a result of decreased level of male sex hormones. In contrast, female tends to have increased asthma prevalence from puberty onset to adulthood mainly due to increased level of female sex hormone. This tendency is reversed during old age mainly due to decreased levels of female sex hormone resulting in a minimal gap between old age male female asthma ratio than the previous stage showing the beneficial role of androgen in old age severe asthma. ↑: minimal increase; ↑↑: high increase; ↑↑↑: highest increase; ↓: minimal decrease; ↓↓: high decrease; ↑↑↓: decrease than previous stage and in decreasing status)

Besides sex hormones, some other factors are also influential in the pathogenesis of childhood asthma. Dysanapsis refers to the differences in airways' growth to lung, and boys are the sufferer of this mechanism as they have smaller airway diameters compared to lung volumes than the girls [28], and females have lower specific lung resistance than males. This phenomenon also makes boys more likely to have asthma episodes than the girls [29]. Obesity is associated with childhood asthma in girls but not in boys [30, 31], and obesity is also associated with hormonal imbalances.

Why do female children experience lower asthma prevalence than the male? Is it anatomical cause or gender differences? Is there any protective effect of female sex hormones for girls or natural selection? Is there any immunologic mechanism that is driving allergic childhood asthma more towards boys? Though the mechanism of age and gender-related asthma differences with hormonal influences is still not precise, we believe the increased prevalence of asthma in male child and shift of prevalence to adult female is strongly associated with the role of sex hormones.

## 4. Menarche, Menstrual Cycle, Sex Hormones, and Asthma

Menarche is the first menstrual cycle that shows the beginning of the onset of reproducing capacity, development of secondary sexual characteristics [32], and beginning in physiological changes in hormonal cycle. The median age of menarche has been decreasing from the last century and is less than 13 years nowadays [33]. Early menarche fluctuates the hormones, increases the asthma incidence [34], and is also associated with increased risk of breast cancer, cardiovascular disease, obesity, and type 2 diabetes [35, 36].

Multiparous women and girls with a history of early menarche have higher exposure to estrogen, increased cumulative exposure to sex hormones, and are at risk of asthma and severe asthma [37, 38]. The twofold increased asthma prevalence in girls with an early history of menarche is possibly due to the role of sex hormones [37, 39, 40]. Low TES levels and premenstrual and menstrual asthma aggravation are correlated in some asthmatic females [41]. Increased adulthood asthma and lower lung function are associated with early menarche, supporting the influence of hormones in women's respiratory health status [42].

Similarly, menstrual cycle worsening of asthma symptoms is linked with the fluctuations of serum levels of estradiol and progesterone [43]. Nevertheless, there is a decrease of provocative concentration of methacholine (PC20) by more than 50% in adult women with stable well-controlled asthma during the menstrual cycle, and the highest decrease occurs in the luteal phase at peak estrogen-progesterone levels [44]. The abnormal *β*2 adrenoceptor regulation is attributed to the cyclic changes in PC20 premenstrual asthma [44, 45].

Two to sevenfold higher serum estradiol and progesterone levels and lower forced expiratory volume in one second (FEV1) and forced vital capacity (FVC) are observed in women with and without asthma during the luteal phase of the menstrual cycle [44, 46]. That is why lower FEV1 and more respiratory symptoms are observed in the premenstrual and menstrual period asthma with an increased level of airway hyperresponsiveness and probably increased medical attendance [44, 47]. Irregular menstruation is also associated with lower lung function [43], and lung function predicts respiratory health. High fractional exhaled nitric oxide (FeNO) correlates with eosinophilic inflammation and increases in women with premenstrual asthma symptoms [48].

Together, these studies indicate the role of circulating estrogen and androgen levels as a reason for more prevalent asthma in females after menarche and during the menstrual and perimenstrual period, but the underlying mechanisms are still unclear.

## 5. Puberty, Sex Hormones, and Asthma

Puberty is a dynamic process of physical changes and sexual maturation regulated by hormones. In boys, adrenal glands produce weaker androgens, and testes increase the production of TES, whereas in girls, the estrogen production is increased from ovaries leading to thelarche, menarche, and adrenal glands that produce androgens such as androstenedione and DHEA leading to pubarche [49].

There is a gender switch in asthma prevalence at puberty from male to female as asthma is more prevalent and severe in female [50, 51]. Fluctuation and surges of endogenous sex hormones are believed to be involved in the gender switch of asthma [52]. The Childhood Asthma Management Program (CAMP) study tracked the progression of asthma and average asthma symptom score in puberty with Tanner scores. At the age of 10 years, the average asthma symptoms increased with the increasing score but started to decline in boys. The score continually increased with the increasing ages in girls [53].

The problem of dysanapsis seems to be corrected with the high levels of TES during puberty, as this hormone induces a more pronounced airway caliber in boys than girls and gives protection from asthma. This may be the reason behind the more prevalent asthma in old age males than women as the hormone level decreases in aged male [28, 54]. In boys, the increase in the dose of PC20 to decrease FEV1 to 20% shows the pubertal improvement in airway responsiveness, but not observed in girls [55], and the PC20 was further increased with sexual maturation in boys [55]. Hormonal fluctuations and correction of anatomical problems are the main influencing factors in the prevalence and gender switch of asthma at puberty.

## 6. Pregnancy, Sex Hormones, and Asthma

Asthma is a common, sometimes fatal medical condition that has a variable impact on pregnancy. Generally, 33% of pregnant women experience worsening asthma symptoms with bronchial hyperresponsiveness, 33% improve, and the remaining one-third have unchanged asthma status [56]. Controlling asthma and maintaining asthma control are vital for reducing fetal and maternal risks linked with asthma during pregnancy. Because poorly controlled asthma and severe asthma exacerbations are associated with increased risk newborn comorbidities like higher rates of prematurity, intrauterine growth retardation, respiratory complications, and hyperbilirubinemia [57], preeclampsia and gestational diabetes are seen on the maternal side. But interestingly, about 7.2% of pregnant women with improved asthma status present with asthma exacerbations [56]. Pregnancy with more severe asthma phenotypes is likely to exacerbate and worsen asthma symptoms frequently [58].

There is a linear increase in asthma prevalence in multiparous women with the increasing order of birth [38], and multiparous women and girls with early menarche are exposed to higher estrogen levels with significant accumulative doses of sex hormones exposure. They tend to have a higher risk of severe asthma [37, 38].

The sex of the fetus is considered a risk factor for worsening asthma symptoms during pregnancy. A female fetus has been linked with worsening maternal asthma symptoms and related to hormonal changes in pregnancy, with the speculation of discharge of fetal sex hormone in the maternal blood resulting in asthma exacerbations, increase in corticosteroid use, and even hospitalization independent of maternal age, smoking status, and body mass index (BMI) [59–62]. Interestingly, female newborns with reduced birthweights were remarkably seen from asthmatic mothers but not in male newborns postulating gender-specific effect [63] and probably male hormonal effect. However, a larger Canadian study did not confirm this finding [64]. The male fetus and increased level of TES might be protective against maternal asthma [62].

It is concluded that pregnancy-induced physiological processes may confound the hormone effect, but mainly hormonal fluctuations either from maternal or fetal side possibly involved in pregnancy-related asthma.

## 7. Adulthood, Middle Age, Sex Hormones, and Asthma

Adulthood is a state where humans are believed to be fully grown and matured with complete physical and intellectual maturity around the completion of second decade. Defining middle age is arbitrary, but it is generally believed to be between 40 and 60 years. This is the age where people start to experience physiological, psychological, and hormonal changes and a somewhat gradual decline in physical activities.

In this age group, asthma is more prevalent in women until the age of 45 years and is also associated with higher mortality [65, 66] and beyond that increased in men suggesting the age-gender interaction [54], with possible hormonal influence. Similarly, women have a higher incidence of nonatopic asthma during the reproductive period, but no gender difference is noted in the incidence of atopic asthma [67, 68].

In females, the transition of lower to higher risk and prevalence of asthma from childhood to adulthood is associated with hormonal transitional fluctuations during normal physiobiological stages like puberty, menarche, menstruation, and menopause [50, 69]. Similarly, the male sex hormone is believed to be involved in the opposite asthma status in males and, overall, suggest sex hormones might have a role in modulating pathways associated with asthma pathogenesis.

## 8. Menopause, Sex Hormones, and Asthma

Menopause is a natural physiobiological process that heralds the penultimate menstrual cycle, generally occurs at late 40s or early 50s, and the decrement of sex hormones usually occurs from this phase. The perimenopausal period is the transition phase from where the asthma in females changes from high incidence occurring in adulthood to low incidence, and less severe from menopause onwards, and is believed to be related to fluctuations in female sex hormones. Age, asthma duration, obesity, HRT, and allostatic load (AL) are the confounding factors that affect the independent effect between hormonal fluctuation and asthma status in menopause.

In postmenopausal women, age-adjusted relative risk of asthma may drop compared with premenopausal women [70, 71], and minimal postmenopausal difference of asthma prevalence is observed between men and women compared to adulthood [50]. The age-adjusted lower relative risk of asthma incidence is noted in postmenopausal women who never received HRT [70], and interestingly, the protective effect of menopause was reversed by estrogen HRT [70].

Overall, the reduced asthma prevalence and severity in postmenopausal women compared to men postulates that asthma improves after menopause, and menopause is the phage of reduced levels of sex hormones that probably have an impact on asthma status changes.

## 9. Old Age, Sex Hormones, and Asthma

The age approaching or exceeding life expectancy is old age, and aging is the process of growing old. The cumulative “wear and tear” on the body due to regular and/or exaggerated response of body physiological systems to environmental challenges is known as AL [72]. It can result in premature ageing [73] and a proper mechanism to understand mortality and morbidity in asthma. AL as early aging is present in the mildest asthma group not receiving ICS [74]. Another aging change associated with asthma is autophagy, a biological process of intracellular degradation associated with conditions like asthma, chronic obstructive pulmonary disease (COPD), cystic fibrosis, pulmonary hypertension, cancer, infection, and even in normal aging. Plasma autophagy marker (LC3B) increases in asthmatics, a more pronounced increase of LC3B seen with severe asthmatics with inverse FEV1 association and direct association with increased age. Eosinophilic persistent asthma has a close correlation with accelerated aging [75]. The immune system and various structural changes occur on aging asthmatics like increased chest wall rigidity, less elastic recoil, and reduced respiratory muscle power that ultimately furnish the declined lung function [76–78] and confound the hormonal impact of asthma.

A noticeable decline in serum TES level is noted with increasing age in an old-aged man [79, 80], resulting in more pronounced and severe asthma prevalence. Low androgen level in childhood and their deficiency in aging males postulate the significant androgen role in childhood and old age asthma [80]. Interestingly, circulating androgens are more related to metabolic risk factors than medical comorbidities in males from young age to middle age to old age, but female sex hormone estrogen is not associated with metabolic risk traits. That is why the association of sex hormones with age, metabolic factors, and medical comorbidities is responsible for alteration in health-related factors [81] and even asthma transition with ages.

Conceivably, low TES in older men possibly exacerbate asthma indicates the association of declining level of sex hormone. It might be used as a potential asthma therapy in old-aged males and possibly even in old-aged females.

## 10. Obesity, Hormonal OCP, Neuronal Factors, Sex Hormones, and Asthma

### 10.1. Obesity, Sex Hormones, and Asthma

Obesity is associated with higher asthma risks, especially in girls and women. Leptin, a key player in body weight regulation, regulates Th1 responses, and the production of asthma-related proinflammatory mediators is associated with obese females in asthma [82]. Regardless of physical fitness, higher asthma prevalence and asthma-related morbidity are seen in girls but not in boys [83], and about five to seven times higher asthma prevalence are seen in obese female above 11 years of age than the normal BMI female [84, 85]. Increasing BMI has also direct association with asthma severity [65]. In contrast, it was interesting that low BMI smoker women are at higher risk of asthma than the high BMI nonsmoker women [86].

Although obese-asthma phenotype gender dimorphism is supported by many reports [55], it is still unclear whether this is caused by gender-specific factors confounded with sex hormones. However, we strongly believe in the role of sex hormones in obesity-mediated asthma.

### 10.2. Hormonal OCP, Sex Hormones, and Asthma

The administration of exogeneous hormones and the development of asthma have been explored in many studies. No significant association is found between OCP and the development of asthma [71]. However, there is a slight significant increase in asthma risk with HRT in women. Recently, it has been found that hormonal contraceptives are beneficial for reproductive-age women and may reduce the risk of severe asthma exacerbations [87]. However, the biological basis of how the hormones influence the asthma outcome needs to be evaluated.

Hormonal contraceptives are beneficial in premenstrual asthma and associated with a lower risk of current asthma and fewer symptoms, (OR 0.68; 95% CI, 0.47–0.98) and (OR 0.68; 95% CI, 0.47–0.98), respectively [37, 88]. The luteal phase sex hormones repression by OCP attenuates the cyclical change in airway hyperresponsiveness as seen through changes in PC 20% to drop FEV1 and PEF [89].

Risk of asthma and wheeze with shortness of breath is increased in menstruating women (OR 1.42; 95% CI, 1.09–1.86) and (OR 1.27; 95% CI, 1.02–1.60) using OCP but not in the women without OCP [38, 90]. However, the risk was not seen in menstruating underweight women [26]. Similarly, OCP is associated with increased wheezing risk in asthmatic women [90, 91] and decreased wheezing in some studies [37, 92]. In 2021, the role of HRT in perimenopausal and postmenopausal women with asthma has been explored, and increased risk of asthma exacerbations in perimenopausal and postmenopausal women is associated with previous history of long-term HRT of at least more than two years but not current [86].

Evidently, hormonal OCP or HRT plays crucial role in asthma pathogenesis and prevalence and influences the outcome. However, the mechanism of how HRT work needs to be evaluated thoroughly.

### 10.3. Neuronal Factors, Sex Hormones, and Asthma

Women with attention deficit hyperactivity disorder (ADHD) reported to have higher prevalence of asthma than men with ADHD which is related to higher prevalence of smoking and obesity [93] and might have a confounding hormonal role.

Studies have shown that, during the decline of estrogen and progesterone, i.e., weeks before menstruation, ADHD symptoms may worsen [94] and improve during elevated estrogen and progesterone, i.e., during pregnancy [95]. However, in another study during the menstrual cycle, enhanced ADHD symptoms were associated with reduced estradiol (E2) levels and increased progesterone or TES levels on the following day [96]. That is the reason while treating the asthmatic female with ADHD, and it is advised to inquire about regularity of the menstrual cycle, hormonal profile, hormone therapy, and other physiobiological stages.

Together, the confounding association between ADHD, obesity, smoking, menstrual cycle, pregnancy, and gender with sex hormones in asthma needs to be thoroughly evaluated.

## 11. Sex Hormones Functional Activities in Asthma

**Figure 3:**
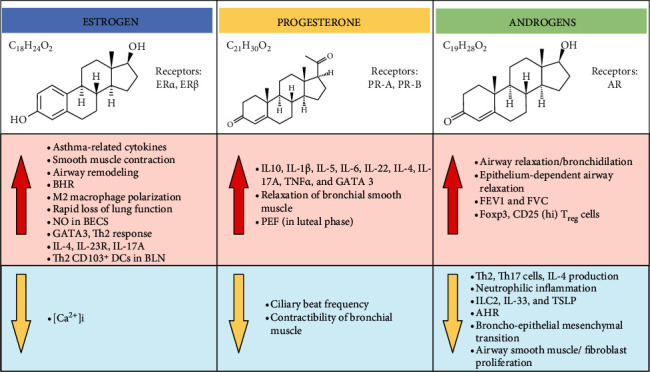
Sex hormones functional activities in asthma. Sex hormones fluctuate and affect asthma progression, remission, or protection by regulating hormonal receptors that act differently. Estrogen mediates its function through the activation of ER*α* and ER*β* receptors. Similarly, PR-A and PR-B are the two isoforms of the progesterone receptor, and androgen exerts its effect through AR. The functions of estrogen, progesterone, and androgen are shown in the figure and explained in Section 11. ER*α*: estrogen receptor alpha; ER*β*: estrogen receptor beta; PR: progesterone receptor; AR: androgen receptor; BHR: bronchial hyperresponsiveness; NO: nitric oxide; BECs: bronchial epithelial cells; [Ca^2+^]i: intracellular ca^2+^ concentration; PEF: peak exploratory flow; FEV1: forced expiratory volume in one second; FVC: forced vital capacity.

Sex hormones are linked with asthma, but the effect and the progression, remission, or protection mechanism of asthma are complex and primarily associated with the hormonal fluctuation [97], mainly through regulating hormonal receptors that act differently. Male gets beneficial protective effect of sex hormones after puberty, and at the same time, girls before puberty and female after menopause have the beneficial effect of sex hormones in asthma. The cyclic changes of hormones are believed to be linked with asthma exacerbations in females [98]. Below, we evaluate the functional status of sex hormones in asthma (Figure 3 for the functional role of sex hormones in asthma).

### 11.1. Estrogens

Estrogen receptor (ERs), important players in allergic lung inflammation, are involved in the increased production of asthma-related cytokines in severe asthma, expressed in majority of immune cells, including macrophages. In asthma, different estrogen receptor *α* (ER*α*) and estrogen receptor *β* (ER*β*) isoforms affect the airway remodeling, lung development, and differential expression of ER*α*, and ER*β* results in airway smooth muscle contraction and changes in intracellular calcium [99]. Exaggerated M2 polarization was observed in an ovariectomized mice; however, after estrogen supplementation and macrophage-specific deletion of ER*α*, it showed impaired M2 polarization [100, 101]. In ASM (airway smooth muscles), ER*β* activation mediates estrogen's proliferation and signaling pathways in asthmatic airway remodeling [102]. Five variants in the ER*α* gene (ESR1) in females are associated with BHR and exaggerated loss of lung function [103].

The fall in circulating estrogen and progesterone correlates with the fall in FEV1 from the luteal phase to follicular phase [46] and accompanies with increased BHR [44] and reduced lymphocyte *β*2 adrenoceptor density [45]. Increased levels of nitric oxide (NO) in the breath generally indicate airway inflammation and asthma. Estrogen increases NO production in bronchial epithelial cells (BECs) of female asthmatics, and its impairment might contribute to altered bronchodilation [104]. In ASM, ER*β* activation reduces (Ca2+)i and is involved in the regulation of ASM contraction in asthma [105]. In asthma, more exaggerated airway remodeling is noted in females, suggesting the involvement of sex hormones and gender factors [106].

A study has shown that estrogen mediates Th2 mediated severe asthma by slight upregulation of the GATA-3 expression and IL-4 production [107]. The 17*β*-estradiol (17*β*-E2) mediated Th2 cytokine production in allergic asthmatic females is attributed to Th2-oriented CD103+ DCs in the BLN (bronchial lymph node) [108].

ER*α* upregulates the IL-23R expression, increases the IL-17A production from Th17 cells, and promotes mitochondrial respiration and proliferation [109]. Similarly, 17*β*-E2 and progesterone together regulate the IL-23R expression partially through let-7f miRNAs and increase the production of IL-17A from Th17 cells in severe asthma [12]. Airway 17*β*-E2 is increased in postmenopausal asthmatic women with severe asthma, and its measurement may have the possibility of a suitable biomarker in diagnosing neutrophils predominant severe asthma [110].

### 11.2. Progesterone

Progesterone exerts its effect through the activation of the progesterone receptors (PRs). PRs expressed in airway epithelium can alter the function of epithelial cells by inhibiting ciliary beat frequency and affect mucociliary clearance during menstrual cycle [111]. PR-A and PR-B are the two isoforms of PRs that exhibit distinctive transcriptional patterns on progesterone response promoters [112]. PR-B is the principal gene transcription activator, while PR-A represses PR-B and ERs transcription [113, 114].

The fall in circulating estrogen and progesterone from the luteal phase to the follicular phase accompanies increased BHR [44] and reduced lymphocyte *β*2 adrenoceptor density [45]. Interestingly, some studies postulated that progesterone increases the relaxation of bronchial smooth muscle and decreases contractility [115] and is considered potent vasodilator in mice pulmonary arteries than estrogen and TES [116]. Luteal phase high progesterone is positively associated with peak expiratory flow rate in women [117]. Improved status of lung function with tissue homeostasis and reduced inflammation is the beneficiary effect of progesterone in the influenza model [118].

Finally, progesterone stimulates the production of proinflammatory cells and upregulates the expression of cytokines and proteins such as IL10, IL-1*β*, IL-5, IL-6, IL-22, IL-4, IL-17A, TNF*α*, and GATA 3 through the regulation of receptors in asthma [12, 107, 118, 119].

### 11.3. Androgens

Androgen receptors (AR) are expressed in many immune cells like B cells, T cells, neutrophils, and macrophages and regulate male sex steroids [120]. AR binds to TES or to active metabolite 5*α*-DHT and becomes activated, acts as a DNA-binding transcription factor via translocating into the nucleus, and controls gene expression. AR regulate the function of immune cells and also affect the lung gene expression. AR upregulation decreases the expression of Th2 and Th17 cells, reduces IL-4 production in lungs, and decreases neutrophilic inflammation in severe asthma [15]. AR share somewhat similar structural features like PRs, high doses of PRs can antagonize AR [121].

The protective effect of male sex hormones in asthma is established in many studies. Asthmatic female possesses higher number of circulating ILC2 in blood, as TES attenuates the function and proliferation of ILC2 in men [122]. TES decreases the expression of ILC2 stimulating cytokines like IL-33 and thymic stromal lymphopoietin (TSLP) and diminishes the ILC2 that ultimately attenuates the airway inflammation [122]. In ILC2 dominant allergic airway inflammation of Rag1−/− mice, increased ILC2-related type 2 inflammation significantly increased in female mice, and TES downregulated the ILC2 and its cytokines suggesting sex differences in ILC2 dominant inflammation and role of androgen therapy in ILC2 dominant asthma [123]. Androgen supplementation seems to be beneficial to reduce airway inflammation and induce airway relaxation in asthmatic women with low androgen level (DHEA − S < 200 *μ*g/dL) [124].

Interestingly, the adrenal androgen DHEA inhibits phosphoinositide 3-kinase–dependent signal pathway and prevents airway remodeling (bronchial epithelial to mesenchymal transition) [125, 126] and also inhibits human ASM and airway fibroblast proliferation during puberty [127, 128]. A significant improvement in lung function is noted with a trial of weak androgen therapy in boys with poorly controlled asthma having low androgen level and showed higher DHEA-S and greater FEV1% predicted are positively correlated [129]. The serum level of TES and DHEA correlate with higher FEV1 and FVC in middle-aged community-dwelling men. That is why androgen might have potential as a biomarker of lung function [130] and suppressive agent in severe asthma.

The protective role of androgens and their effects on T regulatory cells (Tregs) were also studied in asthma. The CD25(hi) Foxp3(1) and IL-10 producing Tregs prevent the Th2 responses to allergens by an increased level of Foxp3 in CD25(hi) Tregs [131]. But in asthma, both Foxp3 expression and CD25(hi) Treg were decreased [132], and this decrease was believed to be due to low androgen level [133]. However, only menstruating women in the ovulatory phase showed increased Foxp3 expression in human CD25(hi) Tregs by androgen and correlated with androgen response element (ARE) within the Foxp3 locus [134].

## 12. MBD2 and Severe Asthma

MBD2 is an important mediator in Th17 predominant neutrophilic severe asthma pathogenesis and is justified by different studies. Our recent study showed the potential role of MBD2 as a novel biomarker for identifying severe asthma various endotypes [135]. Loss of MBD2 tilts towards Th2 polarization by activating dendritic cells for Th2 immunity from CD4 þ T-cell and increases IL-4 and IFN-*γ* expression from CD4+ T cells [7], and MBD2-/- results in a deficiency in Th17 differentiation [136]. Interestingly, MBD2 was found to suppress Treg function through the promotion of Foxp3 demethylation [137]. Below, we review MBD2 and Th17 severe asthma association from different studies (Table 2 shows MBD2 expression and respiratory outcome in asthma).

MBD2 regulates the differentiation of Th17 cells by promoting the gene expression related to the inflammatory process. A study has shown that MBD2 gene silencing significantly lowers the IL-17 expression as increased expression increases the IL-17 expression from splenic CD4+ T cells in an animal model of asthma [138]. The study also postulated MBD2 regulated Th17 differentiation is mediated via affecting the expression of an interferon regulatory factor 4, needed for developing inflammatory Th17 cells in severe asthma [138, 139]. Similarly, the suppressor of cytokine signaling 3 (SOCS3) is a protein involved in negative regulation of STAT3, a Th17 cell differentiation stimulator, which is involved in asthma pathogenesis. In a study, MBD2 downregulated the SOCS3 expression and promoted the differentiation of Th17 cells in severe asthma, showing its involvement in Th17 mediated neutrophilic predominant severe asthma [140].

A transcription factor regulates hypoxia in severe asthma exacerbations and hypoxia-inducible factor-1 (HIF-1), and deficiency of HIF-1*α* reduces Th17 cells development with increased Treg differentiation [141], and study has confirmed that less airway inflammation in HIF-1*α*-/- experimental mice [142] is believed to be due to reduced Th17 cells. MBD2 increases HIF-1*α* in neutrophil-dominant asthma and is involved in increased differentiation and secretion of Th17 cells and IL17 through regulating the HIF-1*α* expression [143] and also reduces the Treg function through an increase in HIF-1*α* suggesting the epigenetic role of MBD2 in severe asthma [141]. Similarly, unsuppressed Tbet/Hlx axis results in deficiency of Th17 cell differentiation in MBD2-/- mice with the protection from experimental autoimmune encephalomyelitis, showing the role of MBD2 in Th17 differentiation [136].

Transforming growth factor-*β*1(TGF *β*1) modulates allergic airway inflammation and remodeling in asthma, correlated with asthma severity, and is probably associated with MBD2 level. MBD2 deficiency attenuates the TGF-*β*1 production with decreased M2 macrophage gathering in bleomycin-induced lung fibrosis [144], and M2 polarized macrophages are correlated with FEV1 in asthma.

It is worth noting that MBD2 is involved in the differentiation and expression of Th17 and its asthma-related inflammatory cytokines via inducing epigenetic changes through different mechanisms, and MBD2 itself and/or MBD2 mediated Th 17 cells can be a potential therapeutic target in severe asthma.

## 13. Therapeutic Possibility of Sex Hormones in MBD2 Mediated Severe Asthma

**Figure 4:**
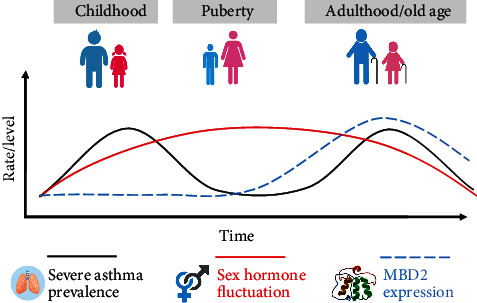
The two peaks of asthma, association, and correlation of sex hormone with MBD2 and Th17 and therapeutic potential of sex hormone in Th17 predominant severe asthma. T2 asthma (T2 high) is early-onset allergic and late-onset nonallergic eosinophilic asthma, sensitive to ICS, and blood eosinophils, FeNO, and IgE levels are common biomarkers [2] and predominantly seen in childhood as the first asthma peak. The second peak is usually adult-onset/old age asthma, non-T2 (T2 low) and is late-onset neutrophilic and paucigranulocytic, as corticosteroids less responsive or resistant, hence also known as severe asthma, and inflammation is driven through T helper 1 (Th1), T helper 17 (Th17) cells, neutrophils, and IL-1ß, IL-8, IL-16, IL-17A/F, IFN-*γ*, and TNF-*α*. [3, 4]. However, these T2 and non-T2 profiles are not always strictly age limited, as children and adults may have both with mixed steroid responses. Adult-onset/old age asthma is generally Th17 predominant, neutrophilic, and severe in nature with MBD2 association [3, 4, 136, 138–144]. The fluctuation curve of sex hormones in the whole life span is shown in the figure. Male sex hormone is beneficial in asthma, as the protective effect from puberty to adulthood reverses in old age due to the reduced androgen level [80]. The expression of MBD2 in humans, as we believe, is shown in figure. It is believed to be at the primary level from birth to adolescence and tends to rise at adulthood, with the plateau probably during old age. At this point, evaluating MBD2, Th17, and the functional role of sex hormones and their correlative association, we believe that male sex hormone regulates the expression of MBD2 and is beneficial in treating Th17 predominant neutrophilic severe asthma of the second peak. ICS: inhaled corticosteroids; FeNO: fractional exhaled nitric oxide; IgE: immunoglobulin E; MBD2: methyl-CpG-binding domain protein 2.

Broadly speaking, we observe the two peaks of asthma in human life [145]. The first peak is during childhood; usually, T2 asthma (T2 high) is early-onset allergic and late-onset nonallergic eosinophilic asthma, sensitive to ICS, and blood eosinophils, FeNO, and IgE levels are the common diagnostic biomarkers [2]. The second peak is usually adult-onset/old age asthma, also known as non-T2 (T2 low) and is neutrophilic and paucigranulocytic, corticosteroids less responsive or resistant, hence also known as severe asthma, and inflammation is driven through T helper 1 (Th1), T helper 17 (Th17) cells, neutrophils, and IL-1ß, IL-8, IL-16, IL-17A/F, IFN-*γ*, and TNF-*α*. Neuronal factors, as well as, comorbidities are also associated in some cases [3, 4]. However, these T2 and non-T2 profiles are not always strictly age limited, mixed steroid responses are also possible, and it is believed epigenetic mechanism is involved in the cellular transformation between the peaks [145] (Figure 4 for two peaks of asthma).

A higher MBD2 level is associated with the pathogenesis of severe asthma (non-T2), as shown from different studies, and MBD2 regulates the expression of Th17 cells in severe asthma [136, 138–144]. Similarly, the increase of male sex hormone is beneficial in asthma, as the protective effect from puberty to adulthood reverses in old age is believed due to the reduced level of androgen [80], and androgen supplementation induces bronchodilatation [146], decreases Th17 (IL17) expression, and reduces the neutrophilic asthma prevalence and exacerbations. This shows the direct correlation of MBD2 with Th17 and an inverse correlation of sex hormones with Th17 and possibly with MBD2. Does androgen downregulate the MBD2 expression in severe asthma (Figure 4)?.

A study has shown that BECs of mice regulate the differentiation of Th2 and Th17 cells [147]. TES relaxes the tracheal smooth muscle [146], but the TES action is annulled with the removal of epithelium, justifying the TES action through the epithelium-dependent manner [148]. This shows that androgen possibly affects downregulation of Th17 via MBD2 in BECs, showing BECs as a cellular target in treating severe asthma. However, we need further cellular studies in this aspect.

Through evaluating different studies and reviewing the association and correlation of MBD2, Th17, and sex hormones in non-T2 asthma, we postulate that male sex hormones possibly downregulate the MBD2 expression resulting in decreased MBD2 mediated Th17 differentiation and might have the therapeutic potential.

## 14. Conclusion and Perspectives

With a better understanding and continuous research on asthma etiology and pathophysiobiology, asthma heterogeneity and endotypes are exclusively explored. T2 asthma, childhood predominant, is better recognized, and several targeted monoclonal antibodies are also identified for therapy. However, non-T2 asthma, usually adult-onset/old age predominant, is still obscure with neutrophilic, paucigranulocytic, or mixed subtypes, where inflammation is driven through varieties of asthma-related inflammatory cells, and with airway muscular, neural, or comorbidities association [3, 4]. Non-T2 asthma is less responsiveness with ICS, hence also called severe asthma. However, these profiles also show mixed steroid responses and are not always strictly age-dependent.

Th17 is the kernel of severe asthma, though proinflammatory cytokines, oxidative stress, and neuronal and hormonal responses, and epigenetic regulation are also involved in the pathogenesis.

MBD2 is an essential mediator of asthma epigenetics because it can bind the target gene, induces posttranscriptional histone modification, interprets DNA methylation, and changes the chromatin structure. MBD2, a Th17 regulator, is involved in the pathogenesis of Th17 predominant neutrophilic severe asthma justified by different studies, and asthma severity and MBD2 expression are correlated. That is why MBD2 has the possibility of a therapeutic target in severe asthma.

Sex hormone fluctuates during the normal physiobiological stages of the human body and affects the asthma outcome. Several internal and external etiopathobiological factors confound the effect of sex hormones and affect the asthma severity, prevalence, remission, and outcome. Male sex steroid tends to show a beneficial effect in severe asthma, and fluctuation is related to asthma prevalence. Boys are at higher risk of asthma prevalence before puberty, but with the increase in androgen level and pronounced airway caliber at puberty onset, male are better protected from asthma until adulthood, as females' prevalence increases at this stage. Later, the tendency of a high prevalence of female adulthood asthma shifts to male at old age due to decreased level of androgen postulating the role of decreased level of male sex hormone in old age Th17 predominant severe asthma.

Is there any downregulatory response on MBD2 by androgens and the role of male sex hormone in MBD2 mediated Th17 predominant severe asthma of old age? This query needs extensive epigenetic and molecular-cellular studies. However, by evaluating the MBD2, Th17, and sex hormone association from various severe asthma studies, the MBD2 expression and Th17 differentiation directly correlate. In contrast, male sex hormone is inversely correlated with MBD2 and Th17 in old age asthma, postulating that the male sex hormone possibly downregulates the MBD2 expression resulting in decreased Th17 differentiation.

Together, given of all studies, we can conclude that evaluation of sex hormone therapeutic potential is a valuable novel approach to solve mysteries surrounding severe asthma therapeutic issues that will guide us towards discovery of new therapeutic agent, reduces the asthma-related complications, and promotes long-term survival that will ultimately reduce the asthma related mortality probably via mechanism distinct from corticosteroid treatment.

## Figures and Tables

**Table 1:** Asthma prevalence and sex hormone cycle in male and female.

	Childhood	Puberty + adolescence	Adulthood	Old age
Male asthma	↑↑	↓	↓↓	↑↑
Female asthma	↑	↑↑	↑↑↑	↑↑↓
Sex hormones	↑	↑↑↑	↑↑	↓

**Table 2:** MBD2 expression and effects on respiratory outcomes.

MBD2	Effects	Respiratory outcome	References
↑ MBD2	↓ Treg function by Foxp3 demethylation	Asthma	[137]
	↑ IL-17 expression	Th17 severe asthma	[138]
	↓SOCS3	Th17 asthma	[140]
	↓ Treg functions	Severe asthma	[141]
	↑ HIF-1*α*	Th17 asthma	[143]

↓ MBD2	↑Th2 polarization	Asthma	[7]
	↑IL-4, IFN-*γ*	Asthma	[7]
	TGF-*β*1 attenuation	↓ airway remodeling, ↓ M2 macrophage gathering	[144]

MBD2-/-	Unsuppressed Tbet/Hlx axis	↓ Th17 differentiation	[136]

## Abbreviations

AR: Androgen receptor
ASM: Airway smooth muscle
BECs: Bronchial epithelial cells
BHR: Bronchial hyperresponsiveness
DHEA: Dehydroepiandrosterone
DHT: Dihydrotestosterone
E2: Estradiol
ERs: Estrogen receptor
FEV1: Forced expiratory volume in one second
FVC: Forced vital capacity
HRT: Hormone replacement therapy
ILC2: Type 2 innate lymphoid cells
MBD2: Methyl-CpG-binding domain protein 2
M2: Macrophage
NO: Nitric oxide
OCP: Oral contraceptive pills
PC20: Provocative concentration 20% fall in FEV1
PRs: Progesterone receptor
TES: Testosterone
Th17: T helper 17 cells
17*β*-E2: 17*β*-estradiol.

## Additional Points

*Key Points*. T2 asthma (T2 high) and non-T2 (T2 low) are the common asthma endotypes. T2 asthma is better known, while non-T2 is still obscure. Non-T2 asthma is generally neutrophilic, paucigranulocytic, or mixed types with a reduced response (resistance) to ICS (that is why also known as severe asthma) and predominantly affects late adulthood and old age population. Th17 is the kernel of severe asthma because of its important involvement in the pathogenesis of severe asthma. Proinflammatory cytokines, oxidative stress, neuronal and hormonal responses, and epigenetic regulation are also involved in Th17 cell-mediated severe asthma, but the underlying mechanisms are not well understood. MBD2 can mediate asthma epigenetics by interpreting DNA methylation and inducing posttranscriptional histone modification through binding the target gene to change the chromatin structure and also affects the Th17 expression in severe asthma, showing the possibility of therapeutic target in severe asthma. Sex hormone fluctuates during the normal physiobiological stages of the human body and affects the asthma severity, prevalence, remission, and outcome. Several internal and external etiopathobiological factors also affect the fluctuation status and function of sex hormones. Boys before puberty and old aged male tend to have higher asthma prevalence, which is related to low and decreased androgen level, respectively. Similarly, girls from puberty to adulthood have higher prevalence of asthma that is associated with their hormonal status. Male sex hormone seems to be beneficial in asthma from above statements. The MBD2 and Th17 expression is directly correlated, while male sex hormone is inversely correlated with MBD2 and Th17 in severe asthma. We expect androgen possibly downregulates the MBD2 expression resulting in decreased Th17 differentiation. Androgen therapy can be valuable novel approach to solve mysteries surrounding severe asthma treatment issues that will guide us towards discovery of new therapeutic agent, reduces the asthma related complications, and promotes long-term survival that will ultimately reduce the asthma-related mortality via novel mechanism distinct from corticosteroid treatment. *Queries for future*. Role of androgen and inhaled/systemic corticosteroid combined therapy in severe asthma. What will be the potential effect of androgen therapy, as androgen itself changes to hormones with estrogenic properties in some extent? What will be the future of triple therapy with sex hormones, corticosteroids, and peroxisome proliferator-activated receptors *γ* (PPAR *γ*) derivatives as they share the near same biochemistry structure?

## References

[1] Patyk M., Obojski A., Gojny Ł., Panaszek B., Zaleska-Dorobisz U. (2016). Airway evaluation with multidetector computed tomography post-processing methods in asthmatic patients. *Advances in Experimental Medicine and Biology*.

[2] Kaur R., Chupp G. (2019). Phenotypes and endotypes of adult asthma: moving toward precision medicine. *The Journal of Allergy and Clinical Immunology*.

[3] Diamant Z., Vijverberg S., Alving K. (2019). Toward clinically applicable biomarkers for asthma: an EAACI position paper. *Allergy*.

[4] Ray A., Kolls J. K. (2017). Neutrophilic inflammation in asthma and association with disease severity. *Trends in Immunology*.

[5] Wood K. H., Zhou Z. (2016). Emerging molecular and biological functions of MBD2, a reader of DNA methylation. *Frontiers in Genetics*.

[6] Du Q., Luu P. L., Stirzaker C., Clark S. J. (2015). Methyl-CpG-binding domain proteins: readers of the epigenome. *Epigenomics*.

[7] Cook P. C., Owen H., Deaton A. M. (2015). A dominant role for the methyl-CpG-binding protein Mbd2 in controlling Th2 induction by dendritic cells. *Nature Communications*.

[8] Trigunaite A., Dimo J., Jørgensen T. N. (2015). Suppressive effects of androgens on the immune system. *Cellular Immunology*.

[9] Kovats S. (2015). Estrogen receptors regulate innate immune cells and signaling pathways. *Cellular Immunology*.

[10] Brabin L. (2002). Interactions of the female hormonal environment, susceptibility to viral infections, and disease progression. *AIDS Patient Care and STDs*.

[11] Tam A., Morrish D., Wadsworth S., Dorscheid D., Man S. F., Sin D. D. (2011). The role of female hormones on lung function in chronic lung diseases. *BMC Womens Health*.

[12] Newcomb D. C., Cephus J. Y., Boswell M. G. (2015). Estrogen and progesterone decrease _let-7f_ microRNA expression and increase IL-23/IL-23 receptor signaling and IL-17A production in patients with severe asthma. *The Journal of Allergy and Clinical Immunology*.

[13] Li Z., Wu F., Brant S. R., Kwon J. H. (2011). IL-23 receptor regulation by let-7f in human CD4+ memory T cells. *Journal of Immunology*.

[14] Laffont S., Blanquart E., Savignac M. (2017). Androgen signaling negatively controls group 2 innate lymphoid cells. *The Journal of Experimental Medicine*.

[15] Fuseini H., Yung J. A., Cephus J. Y. (2018). Testosterone decreases house dust mite-induced type 2 and IL-17A-mediated airway inflammation. *Journal of Immunology*.

[16] Fuseini H., Newcomb D. C. (2017). Mechanisms driving gender differences in asthma. *Current Allergy and Asthma Reports*.

[17] Connelly M. A., Williams D. L. (2003). SR-BI and cholesterol uptake into steroidogenic cells. *Trends in Endocrinology and Metabolism*.

[18] Connelly M. A. (2009). SR-BI-mediated HDL cholesteryl ester delivery in the adrenal gland. *Molecular and Cellular Endocrinology*.

[19] Miller W. L., Auchus R. J. (2011). The molecular biology, biochemistry, and physiology of human steroidogenesis and its disorders. *Endocrine Reviews*.

[20] Russell D. W., Wilson J. D. (1994). Steroid 5 alpha-reductase: two genes/two enzymes. *Annual Review of Biochemistry*.

[21] Di Costanzo L., Drury J. E., Christianson D. W., Penning T. M. (2009). Structure and catalytic mechanism of human steroid 5beta-reductase (AKR1D1). *Molecular and Cellular Endocrinology*.

[22] Caracta C. F. (2003). Gender differences in pulmonary disease. *Mount Sinai Journal of Medicine*.

[23] Bjornson C. L., Mitchell I. (2000). Gender differences in asthma in childhood and adolescence. *The Journal of Gender-Specific Medicine*.

[24] Esposito R., Spaziano G., Giannattasio D. (2019). Montelukast improves symptoms and lung function in asthmatic women compared with Men. *Frontiers in Pharmacology*.

[25] Schatz M., Camargo C. A. (2003). The relationship of sex to asthma prevalence, health care utilization, and medications in a large managed care organization. *Annals of Allergy, Asthma & Immunology*.

[26] Redline S., Gold D. (1994). Challenges in interpreting gender differences in asthma. *American Journal of Respiratory and Critical Care Medicine*.

[27] Melgert B. N., Ray A., Hylkema M. N., Timens W., Postma D. S. (2007). Are there reasons why adult asthma is more common in females?. *Current Allergy and Asthma Reports*.

[28] Townsend E. A., Miller V. M., Prakash Y. S. (2012). Sex differences and sex steroids in lung health and disease. *Endocrine Reviews*.

[29] Pagtakhan R. D., Bjelland J. C., Landau L. I. (1984). Sex differences in growth patterns of the airways and lung parenchyma in children. *Journal of Applied Physiology: Respiratory, Environmental and Exercise Physiology*.

[30] Da Wang Z. Q., Wang J., Yang M. (2014). Gender-specific differences in associations of overweight and obesity with asthma and asthma-related symptoms in 30 056 children: result from 25 districts of northeastern China. *The Journal of Asthma*.

[31] Willeboordse M., van den Bersselaar D. L., van de Kant K. D., Muris J. W., van Schayck O. C., Dompeling E. (2013). Sex differences in the relationship between asthma and overweight in Dutch children: a survey study. *PLoS One*.

[32] Rees M. (1995). The age of menarche. *ORGYN.*.

[33] de Muinich Keizer S. M., Mul D. (2001). Trends in pubertal development in Europe. *Human Reproduction Update*.

[34] Castro-Rodriguez J. A. (2016). A new childhood asthma phenotype: obese with early menarche. *Paediatric Respiratory Reviews*.

[35] Heys M., Schooling C. M., Jiang C. (2007). Age of menarche and the metabolic syndrome in China. *Epidemiology*.

[36] Golub M. S., Collman G. W., Foster P. M. D. (2008). Public health implications of altered puberty timing. *Pediatrics*.

[37] Salam M. T., Wenten M., Gilliland F. D. (2006). Endogenous and exogenous sex steroid hormones and asthma and wheeze in young women. *The Journal of Allergy and Clinical Immunology*.

[38] Jenkins M. A., Dharmage S. C., Flander L. B. (2006). Parity and decreased use of oral contraceptives as predictors of asthma in young women. *Clinical and Experimental Allergy*.

[39] Al-Sahab B., Hamadeh M. J., Ardern C. I., Tamim H. (2011). Early menarche predicts incidence of asthma in early adulthood. *American Journal of Epidemiology*.

[40] Fida N. G., Williams M. A., Enquobahrie D. A. (2012). Association of age at menarche and menstrual characteristics with adult onset asthma among reproductive age women. *Reproductive System & Sexual Disorders*.

[41] Matteis M., Polverino F., Spaziano G. (2014). Effects of sex hormones on bronchial reactivity during the menstrual cycle. *BMC Pulmonary Medicine*.

[42] Macsali F., Real F. G., Plana E. (2011). Early age at menarche, lung function, and adult asthma. *American Journal of Respiratory and Critical Care Medicine*.

[43] Real F. G., Svanes C., Omenaas E. R. (2007). Menstrual irregularity and asthma and lung function. *The Journal of Allergy and Clinical Immunology*.

[44] Tan K. S., McFarlane L. C., Lipworth B. J. (1997). Loss of normal cyclical beta 2 adrenoceptor regulation and increased premenstrual responsiveness to adenosine monophosphate in stable female asthmatic patients. *Thorax*.

[45] Wheeldon N. M., Newnham D. M., Coutie W. J., Peters J. A., McDevitt D. G., Lipworth B. J. (1994). Influence of sex-steroid hormones on the regulation of lymphocyte beta 2-adrenoceptors during the menstrual cycle. *British Journal of Clinical Pharmacology*.

[46] Farha S., Asosingh K., Laskowski D. (2009). Effects of the menstrual cycle on lung function variables in women with asthma. *American Journal of Respiratory and Critical Care Medicine*.

[47] Rao C. K., Moore C. G., Bleecker E. (2013). Characteristics of perimenstrual asthma and its relation to asthma severity and control: data from the severe asthma research program. *Chest*.

[48] Dweik R. A., Boggs P. B., Erzurum S. C. (2011). An official ATS clinical practice guideline: interpretation of exhaled nitric oxide levels (FENO) for clinical applications. *American Journal of Respiratory and Critical Care Medicine*.

[49] Mouritsen A., Søeborg T., Hagen C. P. (2015). Longitudinal changes in serum concentrations of adrenal androgen metabolites and their ratios by LC-MS/MS in healthy boys and girls. *Clinica Chimica Acta*.

[50] Kynyk J. A., Mastronarde J. G., McCallister J. W. (2011). Asthma, the sex difference. *Current Opinion in Pulmonary Medicine*.

[51] Vink N. M., Postma D. S., Schouten J. P., Rosmalen J. G., Boezen H. M. (2010). Gender differences in asthma development and remission during transition through puberty: the TRacking Adolescents' individual lives survey (TRAILS) study. *The Journal of Allergy and Clinical Immunology*.

[52] Zein J. G., Erzurum S. C. (2015). Asthma is Different in Women. *Current Allergy and Asthma Reports*.

[53] Fu L., Freishtat R. J., Gordish-Dressman H. (2014). Natural progression of childhood asthma symptoms and strong influence of sex and puberty. *Annals of the American Thoracic Society*.

[54] Zein J. G., Dweik R. A., Comhair S. A. (2015). Asthma Is More Severe in Older Adults. *PLoS One*.

[55] Tantisira K. G., Colvin R., Tonascia J., Strunk R. C., Weiss S. T., Fuhlbrigge A. L. (2008). Airway responsiveness in mild to moderate childhood asthma: sex influences on the natural history. *American Journal of Respiratory and Critical Care Medicine*.

[56] SCHATZ M., HARDEN K., FORSYTHE A. (1988). The course of asthma during pregnancy, post partum, and with successive pregnancies: a prospective analysis. *The Journal of Allergy and Clinical Immunology*.

[57] Mendola P., Männistö T. I., Leishear K., Reddy U. M., Chen Z., Laughon S. K. (2014). Neonatal health of infants born to mothers with asthma. *The Journal of Allergy and Clinical Immunology*.

[58] Schatz M. (1999). Interrelationships between asthma and pregnancy: a literature review. *The Journal of Allergy and Clinical Immunology*.

[59] Beecroft N., Cochrane G. M., Milburn H. J. (1998). Effect of sex of fetus on asthma during pregnancy: blind prospective study. *BMJ*.

[60] Bakhireva L. N., Schatz M., Jones K. L. (2008). Fetal sex and maternal asthma control in pregnancy. *The Journal of Asthma*.

[61] Kwon H. L., Belanger K., Holford T. R., Bracken M. B. (2006). Effect of fetal sex on airway lability in pregnant women with asthma. *American Journal of Epidemiology*.

[62] Dodds L., Armson B. A., Alexander S. (1999). Use of asthma drugs is less among women pregnant with boys rather than girls. *BMJ*.

[63] Murphy V. E., Gibson P. G., Giles W. B. (2003). Maternal asthma is associated with reduced female fetal growth. *American Journal of Respiratory and Critical Care Medicine*.

[64] Firoozi F., Ducharme F. M., Lemière C. (2009). Effect of fetal gender on maternal asthma exacerbations in pregnant asthmatic women. *Respiratory Medicine*.

[65] (2003). The ENFUMOSA cross-sectional European multicentre study of the clinical phenotype of chronic severe asthma. European network for understanding mechanisms of severe asthma. *The European Respiratory Journal*.

[66] Serra-Batlles J., Plaza V., Morejón E., Comella A., Brugués J. (1998). Costs of asthma according to the degree of severity. *The European Respiratory Journal*.

[67] Beuther D. A., Sutherland E. R. (2007). Overweight, obesity, and incident asthma: a meta-analysis of prospective epidemiologic studies. *American Journal of Respiratory and Critical Care Medicine*.

[68] Ma J., Xiao L. (2013). Association of general and central obesity and atopic and nonatopic asthma in US adults. *The Journal of Asthma*.

[69] Baibergenova A., Thabane L., Akhtar-Danesh N., Levine M., Gafni A., Leeb K. (2006). Sex differences in hospital admissions from emergency departments in asthmatic adults: a population-based study. *Annals of Allergy, Asthma & Immunology*.

[70] Troisi R. J., Speizer F. E., Willett W. C., Trichopoulos D., Rosner B. (1995). Menopause, postmenopausal estrogen preparations, and the risk of adult-onset asthma. A prospective cohort study. *American Journal of Respiratory and Critical Care Medicine*.

[71] Lange P., Parner J., Prescott E., Ulrik C. S., Vestbo J. (2001). Exogenous female sex steroid hormones and risk of asthma and asthma-like symptoms: a cross sectional study of the general population. *Thorax*.

[72] McEwen B. S., Seeman T. (1999). Protective and damaging effects of mediators of stress. Elaborating and testing the concepts of allostasis and allostatic load. *Annals of the New York Academy of Sciences*.

[73] Castagné R., for the Lifepath Consortium, Garès V. (2018). Allostatic load and subsequent all-cause mortality: which biological markers drive the relationship? Findings from a UK birth cohort. *European Journal of Epidemiology*.

[74] Barry L. E., O'Neill C., Heaney L. G. (2020). Association between asthma, corticosteroids and allostatic load biomarkers: a cross-sectional study. *Thorax*.

[75] Belsky D. W., Shalev I., Sears M. R. (2014). Is chronic asthma associated with shorter leukocyte telomere length at midlife?. *American Journal of Respiratory and Critical Care Medicine*.

[76] Enright P. L., Kronmal R. A., Manolio T. A., Schenker M. B., Hyatt R. E. (1994). Respiratory muscle strength in the elderly. Correlates and reference values. Cardiovascular health study research group. *American Journal of Respiratory and Critical Care Medicine*.

[77] Janssens J. P. (2005). Aging of the respiratory system: impact on pulmonary function tests and adaptation to exertion. *Clinics in Chest Medicine*.

[78] Estenne M., Yernault J. C., De Troyer A. (1985). Rib cage and diaphragm-abdomen compliance in humans: effects of age and posture. *Journal of Applied Physiology*.

[79] Harman S. M., Metter E. J., Tobin J. D., Pearson J., Blackman M. R. (2001). Baltimore longitudinal study of aging. Longitudinal effects of aging on serum total and free testosterone levels in healthy men. Baltimore longitudinal study of aging. *The Journal of Clinical Endocrinology and Metabolism*.

[80] Feldman H. A., Longcope C., Derby C. A. (2002). Age trends in the level of serum testosterone and other hormones in middle-aged men: longitudinal results from the Massachusetts male aging study. *The Journal of Clinical Endocrinology and Metabolism*.

[81] Yeap B. B., Knuiman M. W., Divitini M. L. (2014). Differential associations of testosterone, dihydrotestosterone and oestradiol with physical, metabolic and health-related factors in community-dwelling men aged 17-97 years from the Busselton health survey. *Clinical Endocrinology*.

[82] Quek Y.-W., Sun H.-L., Ng Y.-Y. (2010). Associations of serum leptin with atopic asthma and allergic rhinitis in children. *American Journal of Rhinology & Allergy*.

[83] Lu K. D., Billimek J., Bar-Yoseph R., Radom-Aizik S., Cooper D. M., Anton-Culver H. (2016). Sex differences in the relationship between fitness and obesity on risk for asthma in adolescents. *The Journal of Pediatrics*.

[84] Zallo N. A., Aguinaga-Ontoso I., Alvarez-Alvarez I., Guillén-Grima F., Julian C. A. S. (2017). The influence of gender and atopy in the relationship between obesity and asthma in childhood. *Allergologia et Immunopathologia*.

[85] Weiss S. T., Shore S. (2004). Obesity and asthma: directions for research. *American Journal of Respiratory and Critical Care Medicine*.

[86] Nwaru B. I., Shah S. A., Tibble H. (2021). Hormone replacement therapy and risk of severe asthma exacerbation in perimenopausal and postmenopausal women: 17-year National Cohort Study. *The Journal of Allergy and Clinical Immunology. In Practice*.

[87] Nwaru B. I., Tibble H., Shah S. A. (2021). Hormonal contraception and the risk of severe asthma exacerbation: 17-year population-based cohort study. *Thorax*.

[88] Nwaru B. I., Sheikh A. (2015). Hormonal contraceptives and asthma in women of reproductive age: analysis of data from serial national Scottish health surveys. *Journal of the Royal Society of Medicine*.

[89] Tan K. S., McFarlane L. C., Lipworth B. J. (1997). Modulation of airway reactivity and peak flow variability in asthmatics receiving the oral contraceptive pill. *American Journal of Respiratory and Critical Care Medicine*.

[90] Macsali F., Real F. G., Omenaas E. R. (2009). Oral contraception, body mass index, and asthma: a cross-sectional Nordic-Baltic population survey. *The Journal of Allergy and Clinical Immunology*.

[91] Erkoçoğlu M., Kaya A., Azkur D. (2013). The effect of oral contraceptives on current wheezing in young women. *Allergologia et Immunopathologia*.

[92] Dratva J., Schindler C., Curjuric I. (2010). Perimenstrual increase in bronchial hyperreactivity in premenopausal women: results from the population-based SAPALDIA 2 cohort. *The Journal of Allergy and Clinical Immunology*.

[93] Fasmer O. B., Halmøy A., Eagan T. M., Oedegaard K. J., Haavik J. (2011). Adult attention deficit hyperactivity disorder is associated with asthma. *BMC Psychiatry*.

[94] Quinn P. O. (2005). Treating adolescent girls and women with ADHD: gender-specific issues. *Journal of Clinical Psychology*.

[95] Nadeau K., Quinn P., Quinn P. O., Nadeau K. G. (2002). The history of ADHD -- an unexamined gender bias. *Gender Issues and ADHD: Research, Diagnosis and Treatment*.

[96] Roberts B., Eisenlohr-Moul T., Martel M. M. (2018). Reproductive steroids and ADHD symptoms across the menstrual cycle. *Psychoneuroendocrinology*.

[97] Graziottin A., Serafini A. (2016). Perimenstrual asthma: from pathophysiology to treatment strategies. *Multidisciplinary Respiratory Medicine*.

[98] Chandler M. H., Schuldheisz S., Phillips B. A., Muse K. N. (1997). Premenstrual asthma: the effect of estrogen on symptoms, pulmonary function, and beta 2-receptors. *Pharmacotherapy*.

[99] Aravamudan B., Goorhouse K. J., Unnikrishnan G. (2017). Differential expression of estrogen receptor variants in response to inflammation signals in human airway smooth muscle. *Journal of Cellular Physiology*.

[100] Keselman A., Heller N. (2015). Estrogen signaling modulates allergic inflammation and contributes to sex differences in asthma. *Frontiers in Immunology*.

[101] Keselman A., Fang X., White P. B., Heller N. M. (2017). Estrogen signaling contributes to sex differences in macrophage polarization during asthma. *Journal of Immunology*.

[102] Ambhore N. S., Katragadda R., Kalidhindi R. S. R. (2018). Estrogen receptor beta signaling inhibits PDGF induced human airway smooth muscle proliferation. *Molecular and Cellular Endocrinology*.

[103] Dijkstra A., Howard T. D., Vonk J. M. (2006). Estrogen receptor 1 polymorphisms are associated with airway hyperresponsiveness and lung function decline, particularly in female subjects with asthma. *The Journal of Allergy and Clinical Immunology*.

[104] Townsend E. A., Meuchel L. W., Thompson M. A., Pabelick C. M., Prakash Y. S. (2011). Estrogen increases nitric-oxide production in human bronchial epithelium. *The Journal of Pharmacology and Experimental Therapeutics*.

[105] Bhallamudi S., Connell J., Pabelick C. M., Prakash Y. S., Sathish V. (2020). Estrogen receptors differentially regulate intracellular calcium handling in human nonasthmatic and asthmatic airway smooth muscle cells. *American Journal of Physiology. Lung Cellular and Molecular Physiology*.

[106] Takeda M., Tanabe M., Ito W. (2013). Gender difference in allergic airway remodelling and immunoglobulin production in mouse model of asthma. *Respirology*.

[107] Samimi L. N., Fallahpour M., Khoshmirsafa M. (2021). The impact of 17*β*-estradiol and progesterone therapy on peripheral blood mononuclear cells of asthmatic patients. *Molecular Biology Reports*.

[108] Masuda C., Miyasaka T., Kawakami K. (2018). Sex-based differences in CD103+ dendritic cells promote female-predominant Th2 cytokine production during allergic asthma. *Clinical and Experimental Allergy*.

[109] Fuseini H., Cephus J.-Y., Wu P. (2019). ER*α* signaling increased IL-17A production in Th17 cells by upregulating IL-23R expression, mitochondrial respiration, and proliferation. *Frontiers in Immunology*.

[110] Scioscia G., Carpagnano G. E., Lacedonia D. (2020). The role of airways 17*β*-estradiol as a biomarker of severity in postmenopausal asthma: a pilot study. *Journal of Clinical Medicine*.

[111] Jain R., Ray J. M., Pan J. H., Brody S. L. (2012). Sex hormone-dependent regulation of cilia beat frequency in airway epithelium. *American Journal of Respiratory Cell and Molecular Biology*.

[112] Shao R., Egecioglu E., Weijdegard B. (2006). Developmental and hormonal regulation of progesterone receptor A-form expression in female mouse lung in vivo: interaction with glucocorticoid receptors. *The Journal of Endocrinology*.

[113] Lamont K. R., Tindall D. J. (2010). Androgen regulation of gene expression. *Advances in Cancer Research*.

[114] Giangrande P. H., DP M. D. (1999). The A and B isoforms of the human progesterone receptor: two functionally different transcription factors encoded by a single gene. *Recent Prog Horm Res*.

[115] Perusquía M., Hernández R., Montaño L. M., Villalón C. M., Campos M. G. (1997). Inhibitory effect of sex steroids on guinea-pig airway smooth muscle contractions. *Comparative Biochemistry and Physiology. Part C, Pharmacology, Toxicology & Endocrinology*.

[116] English K. M., Jones R. D., Jones T. H., Morice A. H., Channer K. S. (2001). Gender differences in the vasomotor effects of different steroid hormones in rat pulmonary and coronary arteries. *Hormone and Metabolic Research*.

[117] Mannan S. R., Begum N. (2012). Correlation of serum level of progesterone with peak expiratory flow rate (PEFR) in different phases of menstrual cycle. *Anwer Khan Modern Medical College Journal*.

[118] Hall O. J., Limjunyawong N., Vermillion M. S. (2016). Progesterone-based therapy protects against influenza by promoting lung repair and recovery in females. *PLOS Pathogens*.

[119] de Oliveira A. P. L., Peron J. P. S., Damazo A. S. (2010). Female sex hormones mediate the allergic lung reaction by regulating the release of inflammatory mediators and the expression of lung E-selectin in rats. *Respiratory Research*.

[120] Mikkonen L., Pihlajamaa P., Sahu B., Zhang F. P., Jänne O. A. (2010). Androgen receptor and androgen-dependent gene expression in lung. *Molecular and Cellular Endocrinology*.

[121] Kadel S., Kovats S. (2018). Sex Hormones Regulate Innate Immune Cells and Promote Sex Differences in Respiratory Virus Infection. *Frontiers in Immunology*.

[122] Cephus J. Y., Stier M. T., Fuseini H. (2017). Testosterone attenuates group 2 innate lymphoid cell-mediated airway inflammation. *Cell Reports*.

[123] Wang C., Xu Z.-B., Peng Y.-Q. (2020). Sex differences in group 2 innate lymphoid cell-dominant allergic airway inflammation. *Molecular Immunology*.

[124] Marozkina N., Zein J., DeBoer M. D. (2019). Dehydroepiandrosterone supplementation may benefit women with asthma who have low androgen levels: a pilot study. *Pulmonary Therapy*.

[125] Hackett T. L. (2012). Epithelial-mesenchymal transition in the pathophysiology of airway remodelling in asthma. *Current Opinion in Allergy and Clinical Immunology*.

[126] Xu L., Xiang X., Ji X. (2014). Effects and mechanism of dehydroepiandrosterone on epithelial-mesenchymal transition in bronchial epithelial cells. *Experimental Lung Research*.

[127] Koziol-White C. J., Goncharova E. A., Cao G., Johnson M., Krymskaya V. P., Panettieri R. A. (2012). DHEA-S inhibits human neutrophil and human airway smooth muscle migration. *Biochimica et Biophysica Acta*.

[128] Mendoza-Milla C., Jiménez A. V., Rangel C. (2013). Dehydroepiandrosterone has strong antifibrotic effects and is decreased in idiopathic pulmonary fibrosis. *The European Respiratory Journal*.

[129] DeBoer M. D., Phillips B. R., Mauger D. T. (2018). Effects of endogenous sex hormones on lung function and symptom control in adolescents with asthma. *BMC Pulmonary Medicine*.

[130] Mohan S. S., Knuiman M. W., Divitini M. L. (2015). Higher serum testosterone and dihydrotestosterone, but not oestradiol, are independently associated with favourable indices of lung function in community-dwelling men. *Clinical Endocrinology*.

[131] Wan Y. Y., Flavell R. A. (2007). Regulatory T-cell functions are subverted and converted owing to attenuated Foxp3 expression. *Nature*.

[132] Hartl D., Koller B., Mehlhorn A. T. (2007). Quantitative and functional impairment of pulmonary CD4+CD25hi regulatory T cells in pediatric asthma. *The Journal of Allergy and Clinical Immunology*.

[133] Page S. T., Plymate S. R., Bremner W. J. (2006). Effect of medical castration on CD4+ CD25+ T cells, CD8+ T cell IFN-gamma expression, and NK cells: a physiological role for testosterone and/or its metabolites. *American Journal of Physiology. Endocrinology and Metabolism*.

[134] Rothman M. S., Carlson N. E., Xu M. (2011). Reexamination of testosterone, dihydrotestosterone, estradiol and estrone levels across the menstrual cycle and in postmenopausal women measured by liquid chromatography-tandem mass spectrometry. *Steroids*.

[135] Chen Z., Yuan Y., He Y. (2021). MBD2 as a potential novel biomarker for identifying severe asthma with different endotypes. *Frontiers in Medicine*.

[136] Zhong J., Yu Q., Yang P. (2014). MBD2 regulates TH17 differentiation and experimental autoimmune encephalomyelitis by controlling the homeostasis of T-bet/Hlx axis. *Journal of Autoimmunity*.

[137] Wang L., Liu Y., Han R. (2013). Mbd2 promotes foxp3 demethylation and T-regulatory-cell function. *Molecular and Cellular Biology*.

[138] Jia A., Wang Y., Sun W. (2017). MBD2 regulates Th17 cell differentiation and experimental severe asthma by affecting IRF4 expression. *Mediators of Inflammation*.

[139] Brüstle A., Heink S., Huber M. (2007). The development of inflammatory T(H)-17 cells requires interferon-regulatory factor 4. *Nature Immunology*.

[140] Sun W., Xiao B., Jia A. (2018). MBD2-mediated Th17 differentiation in severe asthma is associated with impaired SOCS3 expression. *Experimental Cell Research*.

[141] Shi L. Z., Wang R., Huang G. (2011). HIF1alpha-dependent glycolytic pathway orchestrates a metabolic checkpoint for the differentiation of TH17 and Treg cells. *The Journal of Experimental Medicine*.

[142] Kim S. R., Lee K. S., Park H. S. (2010). HIF-1*α* inhibition ameliorates an allergic airway disease via VEGF suppression in bronchial epithelium. *European Journal of Immunology*.

[143] Xu L., Sun W. J., Jia A. J. (2018). MBD2 regulates differentiation and function of Th17 cells in neutrophils- dominant asthma via HIF-1*α*. *Journal of Inflammation*.

[144] Wang Y., Zhang L., Wu G.-R. (2021). MBD2 serves as a viable target against pulmonary fibrosis by inhibiting macrophage M2 program. *Science Advances*.

[145] Wasti B., Liu S. K., Xiang X. D. (2021). Role of epigenetics in the pathogenesis, treatment, prediction, and cellular transformation of asthma. *Mediators Inflammation*.

[146] Montaño L. M., Flores-Soto E., Sommer B., Solís-Chagoyán H., Perusquía M. (2020). Androgens are effective bronchodilators with anti-inflammatory properties: a potential alternative for asthma therapy. *Steroids*.

[147] Da Liu L. H., Ding N., Sun W. (2019). Bronchial epithelial cells of young and old mice directly regulate the differentiation of Th2 and Th17. *Bioscience Reports*.

[148] Kouloumenta V., Hatziefthimiou A., Paraskeva E., Gourgoulianis K., Molyvdas P. A. (2006). Non-genomic effect of testosterone on airway smooth muscle. *British Journal of Pharmacology*.

